# Differences in severity of reticulo-rumen pH drop in primiparous Holstein cows fed the same diet during transition and early lactation: effects on performance, energy balance, blood metabolites, and reproduction

**DOI:** 10.1093/jas/skae390

**Published:** 2024-12-25

**Authors:** Ezequias Castillo-Lopez, Thomas Hartinger, Mohsen M Farghaly, Nicole Reisinger, Claudia Lang, Laura Klambauer, Johann Huber, Qendrim Zebeli

**Affiliations:** Centre for Animal Nutrition and Welfare, University of Veterinary Medicine Vienna, Vienna, 1210, Austria; Christian Doppler Laboratory for Innovative Gut Health Concepts of Livestock, University of Veterinary Medicine Vienna, Vienna, 1210, Austria; Centre for Animal Nutrition and Welfare, University of Veterinary Medicine Vienna, Vienna, 1210, Austria; Christian Doppler Laboratory for Innovative Gut Health Concepts of Livestock, University of Veterinary Medicine Vienna, Vienna, 1210, Austria; Department of Animal Production, Faculty of Agriculture, Assiut University, Assiut, 71526, Egypt; AHN R&D Center Tulln, dsm-firmenich, Tulln, 3430, Austria; Centre for Animal Nutrition and Welfare, University of Veterinary Medicine Vienna, Vienna, 1210, Austria; Christian Doppler Laboratory for Innovative Gut Health Concepts of Livestock, University of Veterinary Medicine Vienna, Vienna, 1210, Austria; Research and Teaching Farm, VetFarm Kremesberg, University of Veterinary Medicine Vienna, Pottenstein, 2563, Austria; Centre for Animal Nutrition and Welfare, University of Veterinary Medicine Vienna, Vienna, 1210, Austria; Christian Doppler Laboratory for Innovative Gut Health Concepts of Livestock, University of Veterinary Medicine Vienna, Vienna, 1210, Austria

**Keywords:** cattle, lactation, primiparous, ruminal pH, serum metabolites

## Abstract

We recently reported factors leading to different severity of ruminal pH drop in primiparous cows fed the same diet during transition and early lactation. The present study evaluates the effects of those severities on performance and several blood and balance parameters in the same 24 primiparous cows from 3 wk before calving until week 10 in lactation. The dietary concentrate was increased for all cows from 32 before calving to 60% dry matter (**DM)** basis over the first week in lactation, resulting in a diet with 40% non-fiber carbohydrates (**NFC**), and 14.4% physically effective fiber [**peNDF** > 8]. Ruminal pH was monitored with indwelling systems in all cows during the study; then several indices of duration and magnitude of pH change were used as indicators of the severity of ruminal pH drop. Accordingly, as reported in the companion paper, the cows were classified as either higher pH drop (HIGH; *n* = 9), moderate (MOD; *n* = 9), or lower (LOW; *n* = 6) severity of pH drop. In the present report, body weight, body condition score, and back fat thickness decreased but rumen mucosa thickness increased after calving in all cows (*P* < 0.05). Post-partum daily energy intake in HIGH was greater (*P* < 0.05) than in the other categories. In addition, there were 5.6 kg extra of energy-corrected milk in HIGH (*P* < 0.05). Independent of the severity of pH drop, blood glucose, non-esterified fatty acids, beta-hydroxybutyrate, bilirubin, and cortisol were higher, whereas triglycerides, total protein, globulin, albumin, urea nitrogen, cholesterol, calcium, and phosphorous were lower at calving (*P* < 0.05). Blood cholesterol was greater in HIGH than the other pH categories (*P* < 0.05); insulin and reproductive variables were not affected by the severity of the pH drop. Overall, primiparous cows fed the same diet showed different severity of ruminal pH drop, but the individual variation in ruminal pH depression was not related to a negative impact on milk yield, energy balance, or blood metabolites during the study. This indicates that the impact of low ruminal pH on the animals may depend not only on the severity of the pH drop (average of 403 min/d of pH < 5.8 over 13 wk) but also on diet characteristics. Specifically, drops of ruminal pH can be tolerated by primiparous cows during the first 10 wk in milk consuming a diet containing 40% NFC and 14.4% peNDF > 8. It would be worth studying the severity of ruminal pH drop in a larger sample size during the entire lactation and if the severity is sustained on the next lactation.

## Teaser

Primiparous cows show individual differences in the severity of ruminal pH drop. However, these cows seem to tolerate severe drops of ruminal pH during the first 10 wk of lactation in terms of milk production performance, energy balance, and blood metabolites.

## Introduction

The transition of heifers from late gestation to first lactation is characterized by physiologic and metabolic stress, rendering cows vulnerable to health disorders that may impair their production performance and fertility (Esposito et al., 2014). Indeed, the periparturient period in cows is characterized by homeorhetic adaptations involving major shifts in body substrates, which are reflected in significant changes in blood metabolites. For example, decreased insulin responsiveness modifies nutrient partitioning, especially when the energy requirement and glucose demand of the mammary gland are highest (Kenéz et al., 2019). In addition, in a state of negative energy balance, glycogenolysis, and lipolysis are triggered, which reduce body condition score (**BCS**), there is an increase in non-esterified fatty acid (**NEFA**) in blood as well as an increase in incomplete oxidation of NEFA, leading to excessive ketone bodies in blood (Garverick et al., 2013).

To counteract the energy deficit, cows are typically transitioned to starch-rich feeding early after calving, a time when feed intake is also limited. This enhances ruminal fermentation, allowing to harness more glucogenic precursors from feeds, supporting the glucose uptake, and decreasing the energy deficit (Grummer, 1993). However, rapid fermentation and buildup of volatile fatty acids (**VFA)** in the rumen may disrupt the pH regulation (Aschenbach et al., 2011). Ruminal pH is an important parameter for rumen functioning, being commonly used as a biomarker of rumen health. In general terms, a pH below 5.0 (with lactate accumulation higher than 20–40 mM) and clinical signs (e.g., off-feed, lethargy, febrile condition, turgor, rumen hypotonia, and liver damage) are commonly considered as acute acidosis, as commonly reported in feedlot finishing cattle (Owens et al., 1998; Nagaraja and Titgemeyer, 2007). On the other hand, pH values around 5.6 or 5.8 for several hours a day with limited lactate concentrations (<2 mM) and without clear clinical symptoms are common biomarkers of subacute ruminal acidosis (Duffield et al., 2004; Plaizier et al., 2008, 2022). The occurrence of subacute ruminal acidosis has been widely reported (Plaizier et al., 2022), and may develop faster in early-lactation primiparous cows that are naïve to the insult as they cope with homeorhetic adaptations and stress for the first time (Stauder et al., 2020).

Diet (i.e., the balance between readily available carbohydrates and physically effective fiber; **peNDF**) has commonly been seen as the main regulator of the ruminal pH (Zebeli et al., 2008). An increasing body of evidence has also suggested a cow-individual factor for the severity of ruminal pH drop (Gao and Oba, 2014; Humer et al., 2018; Yang et al., 2022; Hartinger et al., 2024). Other studies have also established that this individual severity, despite fed the same diet, leads to lower milk fat content (Nasrollahi et al., 2017), a tendency for low milk fat percent (Khiaosa-ard et al., 2018), or higher proportion in milk fat of C18:1 *trans*-10 as well as the ratio of C18:1 *trans*-10 to C18:1 *trans*-11 (Jing et al., 2018) mainly in cows after the peak of lactation. Specifically for primiparous cows, we have recently reported management and nutritional factors responsible for the severity of ruminal pH drop when cows were fed the same diet during transition and early lactation (Hartinger et al., 2024). Accordingly, in that companion article, data showed that greater severity of pH drop was associated with increased dry matter intake (**DMI)** and major changes in fermentation profile, especially lower acetate, and higher propionate; yet several other parameters of chewing and systemic health were not affected (Hartinger et al., 2024). Higher DMI and greater glucogenic precursors in the rumen of cows with higher severity of the pH drop would imply that these primiparous cows would also have benefitted in terms of blood metabolic profile (i.e., less NEFA and ketones, higher glucose) and milk performance. On the other hand, decreased lipogenic precursors would imply decreased precursors for de novo lipid synthesis in the body and udder. With the same hypothesis, an increased DMI and more depressed ruminal pH would imply an increased passage rate and decline of nutrient digestibility, impairing the nutrient supply of cows, and thus this would decrease the benefits of the increase in DMI and rumen glucogenic precursors.

Therefore, the exact effects of previously classified different severities of pH drop when cows are fed the same diet on blood metabolites and milk production are not clear. Monitoring blood metabolites during the periparturient period contributes to understanding whether primiparous cows differing in severity of ruminal pH drop, and consuming the same diet, cope with the onset of the first lactation. Thus, the objective of this study was to evaluate the response of primiparous cows previously classified with different individual severity of ruminal pH drop, despite being fed the same diet, in terms of lactation performance, blood metabolic indicators of health status, and energy balance from late gestation to early lactation, as well as reproductive variables for the following gestation. We hypothesized that a greater pH drop would offset the benefits of increased DMI and rumen glucogenic precursors in those cows, which would result in no increase in milk yield and no change in blood glucose, though with lower lipogenic precursors (less ruminal acetate) supply for de novo lipogenesis of body fat and in the udder.

## Materials and Methods

The protocol of this study was approved by the institutional animal care and use committee of the University of Veterinary Medicine Vienna and was in accordance with the national authority of Austria according to 26ff of Animal Experiments Act, Tierversuchsgesetz (GZ: 2021-0.009.975).

### Animals and experimental approach

This is part of a larger project; experimental setup, animal feeding and the results for ruminal pH, classification of cows according to severity of ruminal pH drop, DMI, ruminal fermentation, sorting behavior, rumination activity, as well as acute phase proteins, and liver enzymes as indicators of systemic health have been explained in detail earlier (Hartinger et al., 2024). The same cows were also evaluated for claw health before and 3 mo after parturition (Kofler et al., 2023). Before designing the study, we performed a power analysis, whose details are also reported (Hartinger et al., 2024). The power analysis suggested that a statistical test with a 0.05 two-sided significant level would have 90% power to detect an intergroup (low vs. high severity) difference for the minimal detectable difference (**δ**) of daily mean ruminal pH of 30%. The δ of 30% between extreme severity groups was established based on our previous observations of ruminal pH in which cows were also fed a 60% concentrate diet.

Briefly, 24 primiparous Holstein cows were used (699.2 ± 81.5 kg initial body weight [**BW**]). The study started 3 wk before the expected first parturition and ended in week 10 of lactation. Because of differences in calving date, cows were enrolled in the study in a stepwise manner according to the expected calving date. Ruminal pH was monitored every 10 min using indwelling systems (SmaXtec Animal Care GmbH). These pH systems are widely used and have been previously validated (Klevenhusen et al., 2014; Fleming et al., 2021; Studer et al., 2023). In this study, the failure rate observed for the ruminal pH systems was 4%, which was due to sensor failure that stopped recording pH values. This failure rate was comparable to a failure rate of other ruminal pH systems in cattle (Castillo-Lopez et al., 2014). Summarized ruminal pH variables included maximum, mean, minimum, time, and area < pH 5.8, acidosis index calculated as the area < pH 5.8/kg of DMI (Gao and Oba, 2014), and the prevalence of subacute ruminal acidosis episodes. Similar to previous reports (Penner et al., 2007), we used several ruminal pH metrics to categorize the severity of ruminal pH drop in cows. As described in the companion manuscript, a retrospective analysis using the pH parameters collected from the entire experiment confirmed the different degrees of ruminal pH drop among cows, resulting in 3 categories (Supplementary Figure S1). This analysis separated the cows in clusters under the Euclidean distance metric using proc distance and Ward’s minimum variance method with proc cluster in SAS (SAS Institute Inc., Cary, NC). In this study, the term “pH drop” refers to the decline in reticulo-rumen pH that cows experience throughout the study, when fed either the close-up diet or the lactation ration.

The 3 categories of ruminal pH drop classified in the companion paper (Hartinger et al., 2024) were 1) high severity (HIGH, *n* = 9), 2) moderate severity (MOD; *n* = 9), and 3) low severity (LOW; *n* = 6). When feeding the close-up diet, the mean ruminal pH was 6.33, 6.19, and 5.99 ± 0.04 for LOW, MOD, and HIGH, respectively. Whereas when feeding the lactation diet, the mean ruminal pH was 6.29, 6.13, and 5.94 ± 0.04 for LOW, MOD, and HIGH, respectively. When feeding the close-up diet, the time for ruminal pH < 5.8 was 47.5, 80.2, and 355 ± 43.4 min/d for LOW, MOD, and HIGH, respectively. Whereas when feeding the lactation diet, the time for ruminal pH < 5.8 was 75.4, 181, and 446 ± 43.4 min/d for LOW, MOD, and HIGH, respectively (Table 1).

**Table 1.** Ruminal pH variables for each category of ruminal pH and for each of the diets fed to cows during the experiment

	Diet and pH category									
	Close-up			Lactation				*P*-value^1^		
	HIGH	MOD	LOW	HIGH	MOD	LOW	SEM	Diet	Severity	I
pH variable										
Maximum	6.43	6.62	6.73	6.47	6.67	6.75	0.04	0.24	<0.01	0.84
Mean	6.00	6.19	6.28	5.95	6.13	6.27	0.03	0.16	<0.01	0.75
Minimum	5.56	5.75	5.85	5.46	5.63	5.84	0.03	<0.01	<0.01	0.28
Time < 5.8, min/d	301	79.4	48.4	381	186	77.5	37.4	<0.01	<0.01	0.35
Area < 5.8, pH*min/d	44.7	8.74	5.81	57.9	22.6	8.01	5.93	<0.01	<0.01	0.26
Acidosis index^2^	23.6	13.0	9.35	23.9	13.8	5.40	3.46	0.43	<0.01	0.36

^1^ *P*-value for the effect of diet (Diet), the effect of severity of reticulo-rumen pH drop (Severity) and the interaction of Diet and Severity (I).

^2^The duration of ruminal pH < 5.8 divided by dry matter intake.

### Animal housing and maternity care

During this study, cows were housed in a free-stall barn to allow normal herding behavior. The stall was equipped with individual deep litter cubicles with dimensions of 2.6 × 1.25 m, and they were bedded with straw. Additionally, there was an area of ~68 m^2^ with straw bedding, where cows could rest or move freely. This area was located next to the individual cubicles and was close to the automatic feeders, and this space served as the maternity area. Free-choice mineral cubes and clean fresh water were available for ad libitum intake. Cows were visually monitored in person or with remote cameras to detect signs of calving. If signs of difficulty were observed during labor, assistance was provided accordingly. Immediately after parturition, standard operation procedures post-partum were followed, which included physical examination, rehydration with energy drink supplement containing electrolytes, dextrose, and calcium (Rindavital Energietrunk, H.Wilhelm Schaumann GmbH & Co KG, Brunn am Gebirge, Austria), as well as first milking.

### Diet formulations and feeding

The diets were formulated for Holstein cows in either late pregnancy or early lactation with 650 kg BW, 35 kg milk yield containing 4.0% fat, and 3.6% protein; this was performed following the nutritional recommendations of the Society of Nutrition Physiology (GfE, 2001). Three weeks before the expected parturition, cows were offered the same close-up diet, which contained on **DM** basis 38% grass silage, 30% corn silage, and 32% concentrate for close-up heifers. The chemical analyses of diets were conducted at the University of Veterinary Medicine Vienna through wet chemistry according to the VDLUFA (2012) guidelines as detailed in (Hartinger et al., 2024). Briefly, these analyses included DM (method 3.1), ash (method 8.1), crude protein (method 4.1.1), ether extract (method 5.1.2), neutral detergent fiber (**NDF)** (method 6.5.1), ADF (method 6.5.2), and starch using a commercial kit (Megazyme, Wicklow, Ireland). According to the chemical composition analyses, this diet resulted in (DM basis) 15.2% crude protein (**CP)**, 41.1% NDF, 35.7% non-fiber carbohydrates (**NFC**), 25.4% physically effective NDF > 8 mm (peNDF_>8_), and 6.27 MJ/kg DM net energy for lactation (**NE**_**L**_). After parturition, cows were transitioned to a diet containing 24% grass silage, 16% corn silage, 60% DM concentrate for lactating cows, resulting in a diet with (DM basis) 16.7% CP, 34.3% NDF, 40.2% NFC, and 14.4% peNDF_>8_ (Table 2). The dietary change was performed through gradual daily increments of concentrate over the first week of lactation. This dietary change represented an increase of 1.5%, 4.5%, and 0.3 MJ/kg DM for protein, NFC, and NE_L_, respectively, compared to the close-up diet.

**Table 2.** Ingredients, chemical composition, particle size fractions, and physically effective fiber of the pre-partum and lactation diets fed to cows during the study

	Diet	
Item	Close-up	Lactation
Ingredients (% DM basis)		
Grass silage	38.0	24.0
Corn silage	30.0	16.0
Close-up diet concentrate^1^	32.0	0
Lactation diet concentrate^2^	0	60.0
TMR chemical composition^3^		
DM, % as fresh	46.6 ± 5.10	48.8 ± 4.19
Crude protein, %	15.2 ± 0.78	16.7 ± 1.04
Neutral detergent fiber, %	41.1 ± 2.99	34.3 ± 3.64
Acid detergent fiber, %	26.1 ± 0.87	20.8 ± 3.78
Starch, %	18.1 ± 1.93	25.0 ± 2.46
Non-fiber carbohydrates, %	35.7 ± 2.67	40.2 ± 3.40
Residual OM, %	17.6 ± 1.00	15.2 ± 2.35
Ether extract, %	2.29 ± 0.21	2.29 ± 0.46
Ash, %	7.10 ± 0.13	6.94 ± 0.58
Particle fraction (% retained)^4^		
Long	19.2	10.4
Medium	42.5	31.5
Short	35.3	51.3
Fine	2.98	6.83
Physical effectiveness factor	0.62	0.42
Physically effective NDF_>8 mm_	25.4	14.4
NE_L_, MJ/kg DM^5^	6.2	6.6

^1^Close-up: the concentrate mixture contained: barley meal (62.4%), rapeseed meal (20.0%), corn meal (9.0%), soybean meal (4.0%), Biomin vital dry (3.5%), molasses (1.0%), and magnesium oxide (0.1%).

^2^Lactation: the concentrate mixture contained: barley meal (61.5%), rapeseed meal (16.0%), corn meal (10.00%), soybean meal (8.0%), Biomin M 18 (2.8%), molasses (1.0%), limestone (0.6%), and magnesium oxide (0.07%).

^3^Values for chemical composition of the close-up diet represent the average of 5 samples (1 sampling point for each of the 5 groups of cows); whereas for the lactation diet, values represent the average of 20 samples (4 sampling points for each of the 5 groups of cows).

^4^Particle fractions determined with the Penn State Particle Separator with a 19-mm screen (long), 8-mm screen (medium), 1.18-mm screen (short), and a pan (fine), (Kononoff et al., 2003).

^5^Based on estimated net energy value of each feed and their rate of inclusion in the ration formulation program.

Before the initiation of the experiment, cows were randomly assigned to automatic feeders, so that each cow was allowed access to one independent feeder throughout the experiment using an ear tag transponder (Insentec B. V., Marknesse, Netherlands). Thus, the rations were independently given to each cow, and measurements of feed intake and related data were collected individually. The total mixed rations (**TMR**) were prepared daily in the morning using a feed mixer (Trioliet Triomatic T15, Oldenzaal, Netherlands) and were offered as a TMR in the automatic feeders at 0700 hours. To ensure cows had ad libitum access to feed, feeders were refilled at 1600 hours when needed to target 5%–10% refusals each day. Before the morning feeding, refusals were collected, weighed, and feed bins were cleaned every day. The daily DMI was averaged on a weekly basis.

### Milking and milk sample collection and analysis

Cows were milked using a double-4 tandem parlor (DeLaval GmbH, Eugendorf, Austria) at 0600 and 1700 hours. Milk production was recorded on a daily basis by an electronic machine recorder (DeLaval Corp., Tumba, Sweden). The energy corrected milk (ECM) (kg/d) was calculated as follows: (0.38 × milk fat percentage + 0.21 × milk protein percentage + 0.95) × kg of milk/3.2 (GfE, 2001). Milk samples were collected during the a.m. and p.m. milking every other week from week 2 until week 10 of lactation. Milk samples were preserved with 2-bromo-2-nitropropane-1,3-diol and were temporarily stored in a fridge at 4 °C. Then, samples were analyzed with near-infrared spectroscopy for protein, fat, lactose, and milk urea nitrogen (**MUN**). In addition, somatic cell count (**SCC**), pH, fat-free DM, were measured at Landeskontrollverband Lower Austria, Austria (www.lkv-service.at).

### Feed sampling and analysis

Samples of each feed ingredient as well as samples of the TMR were collected 3 wk before the expected calving date, then in week 1, 4, 7, and 10 of lactation. Feed samples were stored at 20 °C before chemical analyses were performed. Briefly, all analyses were conducted according to official protocols (VDLUFA, 2012), which included ash through combustion overnight at 580 °C CP following the Kjeldahl method, ether extracts using the Soxhlet extraction system (Extraction System B-811, BÜCHI Corporation, Flawil, Switzerland), the ash-free neutral detergent fiber (**aNDFom**) and ash-free acid detergent fiber (**ADFom**) contents with sodium sulfite and reported exclusive of residual ash (Gerhardt GmbH & Co. KG), and with heat-stable α-amylase for aNDFom. Starch content was measured with the K-TSTA kit (Megazyme Ltd., Bray, Ireland). NFC was calculated as 100 − (% CP + % NDF + % ether extract + % ash); residual organic matter (**OM**) was calculated by partitioning the NFC into starch and residual OM (Weiss and Tebbe, 2019).

Particle size distribution of TMR was measured using a Penn State Particle Separator (**PSPS**) equipped with 3 screens (19.0, 8.0, and 1.18 mm) and a pan (Kononoff et al., 2003). To improve accuracy in the measurement of peNDF > 8 mm, pellets that remained on the 8-mm screen were manually transferred to the lower pan of the PSPS. The peNDF and physical effectiveness factor (**pef**) were calculated as outlined elsewhere (Beauchemin and Yang, 2005). The peNDF content of the diet was determined by multiplying the NDF content of the diet by its pef, which was calculated as the sum of the proportion of particles retained on the 19.0- and 8.0-mm sieves.

### Measurements of BW and BCS

BCS (1–5 scale) and BW were measured on days 21 and 7 relative to expected parturition; then, on days 14, 28, 42, 56, and 70 post-partum. These measurements were taken in the morning before feeding. Metabolic body size was calculated as BW^0.75^ (NASEM, 2021). The BCS of cows was measured by a single, trained individual, and the scoring method used was similar to that reported by other researchers (Wildman et al., 1982), but reported to the quarter point.

### Back fat and RMT

Back fat thickness (**BFT**) was measured on day −21 relative to expected parturition; then, measurements were performed on days 42 and 70 of lactation. The transducer was positioned vertically to an imaginary line between the hooks and pins at the sacral examination site. It was held lightly and orthogonal to the interface of fat and muscle while cows being in normal and relaxed posture. These measurements were conducted using a portable B-mode ultrasound generator (SonoVet 600V; BCF Technology Ltd., West Lothian, UK) with a linear transducer and a frequency of 5.0 MHz (Schröder and Staufenbiel, 2006). Likewise, rumen mucosa thickness (**RMT**) was measured with ultrasound on the same days as BFT evaluations using a frequency of 6.0 MHz. This was conducted similarly to the approach followed by other researchers (Mirmazhari-Anwar et al., 2013; Neubauer et al., 2018). To do so, after clipping and cleaning the area for analysis, the RMT measurement was conducted in triplicate, and the values were averaged. The transducer was positioned at the intercept of the lines from the third lumbar vertebra and costal-bone-cartilage border. These measurements were performed by a single, trained individual.

### Collection of blood samples and analyses

Blood samples were collected from the jugular vein on days 21 and 7 relative to the expected parturition. After parturition, collections were performed on days 1, 3, 7, 10, 14, 21, 28, 42, 56, and 70 of lactation. The blood sample collection for day 1 was performed immediately after parturition (approximately within 50 min post-calving). Samplings for the other time points were conducted before the morning meal. After collection, blood serum samples were stored at room temperature for ~1.5 h. Blood samples used for glucose analysis were placed in tubes containing sodium fluoride and were stored on ice or in a fridge for 1.5 h. Then, all tubes were centrifuged at 2,000 × *g* at 4 °C for 15 min (Centrifuge 5804 R, Eppendorf; Hamburg, Germany), the supernatant was pipetted into 2-mL tubes (Eppendorf) and stored at −80 °C.

Analyses of blood metabolic profile were conducted after completion of the experiment. Briefly, kits from Roche Diagnostics (Basel, Switzerland) were used to measure blood components; namely glucose (kit glucose HK, 20767131322), total proteins (kit total protein Gen.2, 03183734190), albumin (kit albumin Gen.2, 03183688122), blood urea nitrogen (**BUN**, kit Urea/BUN, 04460715190), triglycerides (kit triglycerides, 20767107322), NEFA (kit NEFA, WA434-91795 + WA436-91995), beta-hydroxybutyrate (**BHB**) (kit D-3-Hydroxybutyrate, RB 1007), cholesterol (kit cholesterol Gen.2, 03039773190), calcium (kit calcium Gen.2, 05061482190), and phosphorous (kit phosphate inorganic ver.2, 03183793122). In addition, the concentration of blood progesterone was assessed (kit immulite 2000, Siemens, Munich, Germany). These were performed at the Central Clinical Pathology Unit, University of Veterinary Medicine (Vienna, Austria) with an automated autoanalyzer (Cobas 6000/c501; Roche Diagnostics GmbH, Vienna, Austria). Furthermore, ELISA kits were used to determine blood concentrations of cortisol (DEH3388, Version 2-02/17, Demeditec Diagnostics GmbH, Kiel, Germany) and insulin (31-3162, Version 7.0, Mercodia AB, Upssala, Sweden). Blood globulin content was estimated as the difference between total proteins and albumin (Grünberg et al., 2011). The intra-assay variation was controlled by limiting the coefficient of variation to <5% for the blood variables.

### Post-partum monitoring of cows, insemination, and reproduction data

Regarding the second gestation, cows were closely monitored visually as well with the use of Smartbow systems for heat detection (https://www.tech2b.at/startup/smartbow-gmbh/). No estrus synchronization was implemented. Following recommendations of the Smartbow system; artificial insemination was performed between 12 and 18 h after heat was first detected. Pregnancy tests were performed through rectal palpation as well as with ultrasound around 6 and 7 wk after insemination. The interval of calving to first service, the number of inseminations until successful pregnancy, the calving to conception interval, and the calving interval were recorded.

### Estimations of energy and nitrogen balance

Energy balance was evaluated as the difference between energy intake and energy expenditure or output. To do so, daily total energy intake was calculated from values of feed intake and corresponding estimate of the energy content of the rations. The NE_M_ was calculated as 0.293 × BW^0.75^. Energy output from milk was calculated based on milk yield, ECM × 3.14. Energy expenditure for pregnancy in the last 3 wk before parturition was considered to be 18 MJ NE_L_/d as recommended (GfE, 2001).

Nitrogen balance was calculated using values of the nitrogen content of feed, feed intake as well as estimated nitrogen excretion or utilization for pregnancy (NASEM, 2021; Spanghero and Kowalski, 2021). Specifically, nitrogen output from milk was estimated taking into account milk yield and milk nitrogen. The fecal and urine nitrogen output (g/d) was calculated as: 2.16 + (0.631 × X) − (12.8 × Y) + (0.392 × Z) + (4.81 × A) + (0.137 × B) + (0.426 × C), where

X = Nitrogen intake in g/d,

Y = ME in MJ/kg DM of feed,

Z = % NDF in diet,

A = % CP in diet,

B = dry matter intake,

C = BW^0.75^ in kg.

The nitrogen utilization for pregnancy was calculated as: MP pregnancy: ([0.69 × days in pregnancy − 69.2] × [calf birth weight/45])/0.33, where the value 0.33 represents the efficiency of MP utilization for pregnancy (NASEM, 2021).

### Statistical analysis

The ruminal pH data collected every 10 min for each cow were used to calculate daily ruminal pH variables in Microsoft excel (minimum pH, mean pH, maximum pH, duration below pH 5.8, area below pH 5.8, and acidosis index). Normal distribution was checked with proc univariate (SAS Institute Inc.) using the Shapiro-Wilk method. Data were checked for the presence of outliers using Cook’s distance (Cook, 1977), which were removed. Reproduction data were also analyzed for normal distribution, which indicated a lack of normality in these variables. After the outlier check and removal, if the normality was not met, data transformation was performed (log or square root). For the variables duration below ruminal pH 5.8, area below ruminal pH 5.8, and acidosis index, square root transformation was used. The variables that did not require transformation were maximum ruminal pH, mean ruminal pH, and minimum ruminal pH because they met normality after outlier removal. Other than that, log transformation was used for the rest of the variables. The analysis of variance was performed before and after data transformation using proc mixed. For the transformed variables, the LS mean and standard error reported in Tables and Figures are those obtained before transformation (rather than performing back-transformations). The statistical model included the severity of ruminal pH drop and sampling time point (day or week relative to calving) as fixed effects. In addition, the model included cow as a random effect. Cow was used as the experimental unit. To compare the ruminal pH between the pre-partum and lactation diets, the statistical model included the fixed effects of the severity of ruminal pH drop and diet as well as their interaction; cow within diet was included as a random effect. Data from different time points from the same cow in the same treatment were processed as repeated measures with first-order variance-covariance (AR[1]) or compound symmetry (**CS**) structure matrices, considering that the variance-covariance decays with time. The PDIFF option was also included, which allowed multiple comparisons of means throughout the evaluated time points. The Tukey-Kramer method was used for post hoc adjustment. Additionally, the orthogonal contrast between HIGH and LOW cows, as well as the contrast between HIGH and MOD + LOW cows were evaluated. Significance was declared when *P* ≤ 0.05 and a tendency is indicated if 0.05 < *P* ≤ 0.10.

## Results

During the study, cows showed good health and no clinical health issues requiring veterinary treatment were found that may be reflected in reproduction performance, feed intake, rumen fermentation, lying behavior, or claw health, thus none of them required veterinary treatment.

### Energy and nitrogen intake, and milk production performance

During the close-up period, there was a decrease in daily energy intake from 79.8 to 70.6 MJ of NE_L_ (*P* < 0.05), but with no effect of severity of ruminal pH drop (*P* = 0.93).

After parturition (Table 3), there was a gradual increase in average daily energy intake. Cows with a HIGH severity of pH drop had greater daily energy intake compared to both MOD and LOW cows (14.7 MJ of NE_L_ greater, *P* < 0.05). Additionally, cows with a HIGH severity of pH drop had greater nitrogen intake (*P* < 0.05).

**Table 3.** Dry matter intake and milk production performance of primiparous Holstein cows with different severity of ruminal pH drop

	Week within the experiment and ruminal pH drop severity^1^																		
	Week 2			Week 4			Week 6			Week 8			Week 10				*P*-Value		
	HIGH	MOD	LOW	HIGH	MOD	LOW	HIGH	MOD	LOW	HIGH	MOD	LOW	HIGH	MOD	LOW	SEM	Week	Severity	I^2^
DMI, kg/d	13.9	11.4	10.9	18.1	14.9	14.6	19.8	15.8	16.3	19.7	16.6	18.5	21.6	19.5	20.3	0.99	<0.01	< 0.05	<0.05
N intake, g/kg BW^0.75^	2.94	2.52	2.26	3.85	3.32	3.07	4.19	3.50	3.37	4.12	3.68	3.83	4.40	4.17	4.17	0.23	<0.01	<0.05	0.71
Energy intake, MJ NE_L_/d	88.2	77.4	75.4	115	97.4	100	128	107	103	128	108	121	141	131	133	7.01	<0.01	<0.05	0.32
Energy intake, MJ NE_L_/kg BW^0.75^	0.72	0.62	0.56	0.94	0.82	0.77	1.03	0.87	0.84	1.01	0.91	0.96	1.11	1.05	1.04	0.05	<0.01	0.07	0.49
Milk, kg/d	33.4	27.2	29.6	37.9	31.8	35.0	41.2	31.8	33.0	42.8	35.6	34.3	43.4	35.2	38.0	2.35	<0.01	<0.01	0.07
ECM^3^	28.2	25.1	28.2	28.2	26.3	30.9	42.7	32.4	27.5	40.7	32.4	32.4	38.9	30.9	34.7	1.10	<0.01	0.06	<0.05
Milk eff^4^	2.26	2.32	2.60	1.57	1.83	2.01	2.27	2.18	1.80	2.19	2.01	1.80	1.86	1.65	1.76	0.29	<0.01	0.97	0.19
Fat, %	2.95	3.94	3.64	2.42	2.66	3.44	4.01	4.01	2.89	3.75	3.52	3.66	3.33	3.15	3.47	0.51	0.30	0.84	0.48
Fat, kg	0.97	0.91	1.07	0.88	0.81	1.08	1.66	1.35	0.94	1.62	1.23	1.25	1.45	1.11	1.31	0.26	<0.01	0.22	0.25
Protein, %	3.56	3.58	3.56	3.15	3.27	3.25	3.07	3.16	3.21	3.15	3.16	3.27	3.21	3.29	3.24	0.12	<0.01	0.80	0.87
Protein, kg	1.17	1.00	1.04	1.20	1.04	1.12	1.27	0.99	1.15	1.35	1.12	1.12	1.40	1.16	1.23	0.06	<0.01	<0.01	0.49
Fat/protein	0.90	1.04	1.10	0.82	0.54	1.09	1.67	1.23	0.93	1.15	1.17	1.15	1.03	0.93	1.15	0.24	0.06	0.49	0.20
Lactose, %	5.01	4.80	4.68	5.00	5.02	5.06	4.89	4.93	5.07	5.00	4.95	5.02	5.00	5.00	4.95	0.07	<0.01	0.65	<0.05
Lactose, kg	1.66	1.40	1.50	1.85	1.60	1.69	2.00	1.54	1.64	2.13	1.74	1.70	2.16	1.74	1.87	0.07	<0.01	<0.05	0.46
MUN, mg/dL	17.7	21.6	18.1	17.5	15.5	14.4	19.5	24.4	27.5	16.4	22.0	24.6	19.1	22.7	10.5	4.88	0.08	0.54	0.24
pH of milk	6.61	6.67	6.50	6.64	6.65	6.63	6.57	6.70	6.69	6.63	6.68	6.62	6.60	6.66	6.60	0.04	0.23	<0.05	<0.05
Cell count,	24.0	40.3	22.7	27.6	13.2	45.6	42.6	33.8	12.3	30.7	34.0	41.5	31.2	19.8	33.1	1.66	0.92	0.71	0.59
Fat-free DM	9.32	9.12	8.95	8.96	8.99	9.02	8.66	8.79	8.97	8.85	8.82	9.00	8.90	8.98	8.89	0.16	<0.01	0.97	<0.05

Abbreviation: MUN = Milk urea nitrogen.

^1^HIGH: high severity of ruminal pH drop; MOD: moderate severity of ruminal pH drop; and LOW: low severity of ruminal pH drop.

^2^Interaction between severity of ruminal pH drop and week of the experiment.

^3^Energy corrected milk.

^4^Milk yield efficiency was calculated as energy-corrected milk divided by dry matter intake.

Cows with HIGH severity of ruminal pH drop had greater milk yield (6.5 kg higher) and ECM (5.6 kg higher) compared to both MOD and LOW cows (*P* < 0.05; Table 2), which represented an average availability of 17.5 MJ NE_L_/d. The greater yield of ECM was particularly clear from week 4 of lactation onwards. Additionally, although the percent of milk components was not affected by the severity of ruminal pH drop, milk fat yield (*P* = 0.09), protein yield (*P* < 0.01) as well as lactose yield (*P* < 0.05) were 0.19, 0.18, and 0.32 kg/d greater for HIGH cows compared to both MOD and LOW cows. The milk efficiency, MUN, and SCC were not affected by either week of lactation or the severity of the ruminal pH drop. In week 6 of lactation, HIGH cows had lower milk fat-free DM compared to LOW cows (*P* < 0.05).

### Body Weight, Body Condition Score, Back Fat Thickness, and Rumen Mucosa Thickness

**Figure 1.**
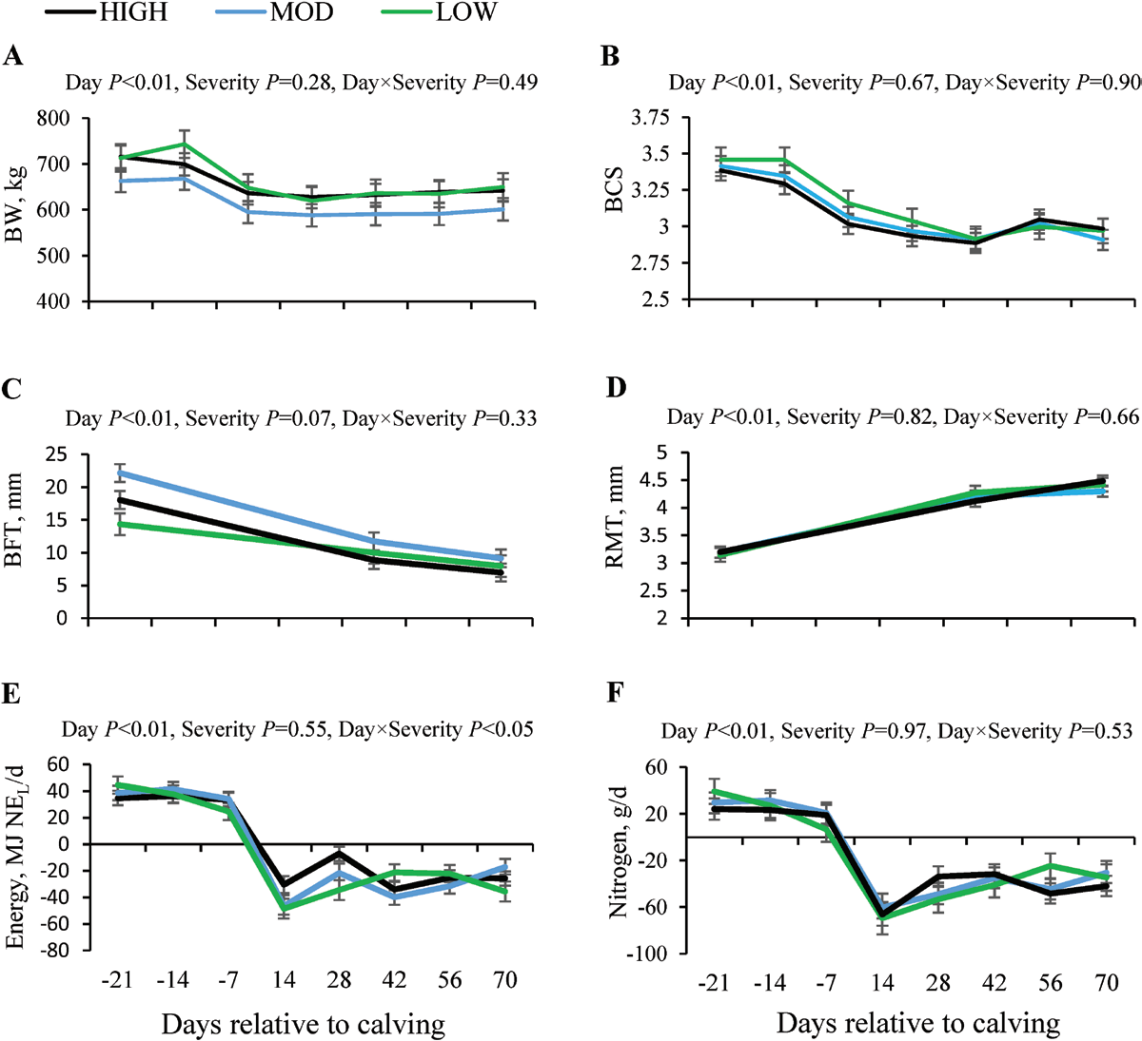
Changes in body weight (BW, A), body condition score (BCS, B), back fat thickness (BFT, C), rumen mucosa thickness (RMT, D), energy (E), and nitrogen balance (F) relative to calving in primiparous Holstein cows with different severity of ruminal pH drop. Abbreviations: HIGH, high severity; LOW, low severity; MOD, moderate severity.

BW, BCS, and BFT declined with the transition from pre- to post-partum (*P* < 0.01). On the other hand, RMT was greater by 1.1 mm in lactating cows compared to values measured before the start of lactation (*P* < 0.01). There was no difference in BW, BCS, and RMT due to the severity of the ruminal pH drop (Figure 1A–D).

### Energy and nitrogen balance

The drop in energy balance with the transition from the dry period to early lactation reached lowest in week 2 of lactation (*P* < 0.05). However, this drop in energy balance was less in HIGH cows (a change of 63 MJ NE_L_/d) compared to MOD and LOW cows (an average change of 77 MJ NE_L_/d, Figure 1E). The nitrogen balance declined at the start of lactation (*P* < 0.01), reaching lowest values in week 2 of lactation (~−70 g/d), but without an effect of severity of ruminal pH drop (Figure 1F).

### Blood metabolic profile

**Figure 2.**
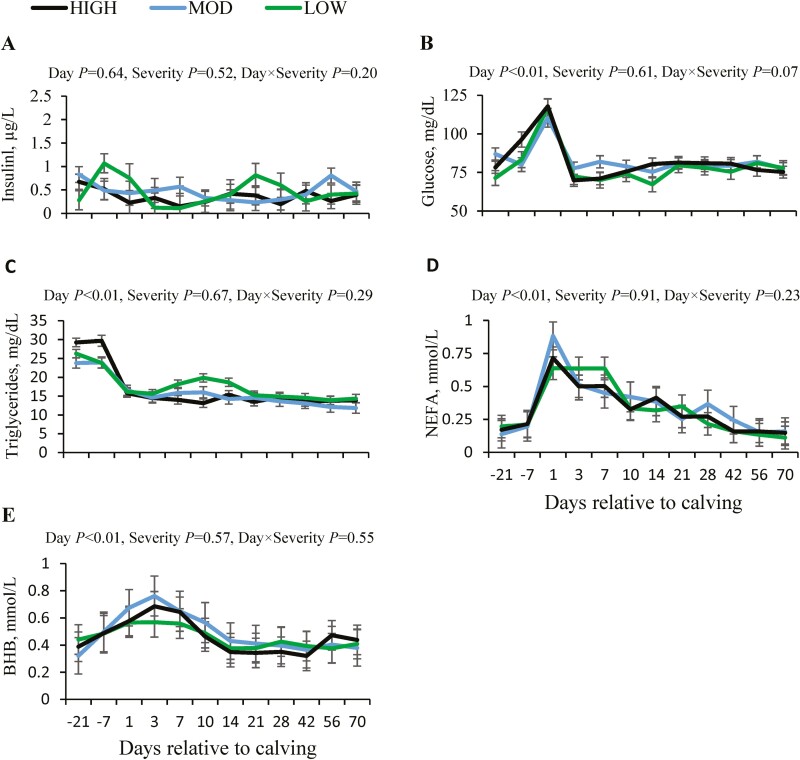
Changes in blood concentrations of insulin (A), glucose (B), total triglycerides (C), non-esterified fatty acids (NEFA, D), and beta-hydroxybutyrate (BHB, E) relative to calving in primiparous Holstein cows with different severity of ruminal pH drop. Abbreviations: HIGH, high severity; LOW, low severity; MOD, moderate severity.

Blood insulin levels were not affected by timepoint relative to calving (*P* = 0.64) or by the severity of pH drop (*P* = 0.52; Figure 2A). However, blood glucose concentrations showed an increase at calving but decreased on day 3 post-partum (*P* < 0.01), and this level was maintained until day 70 of lactation (Figure 2B). No effect of severity of ruminal pH drop was found on blood glucose (*P* = 0.61).

Blood concentration of triglycerides declined at calving (*P* < 0.01), without an effect of severity of ruminal pH drop (*P* = 0.67, Figure 2C). However, blood NEFA increased at calving (*P* < 0.01); then, NEFA started to decline so that on days 56 and 70 of lactation these values were similar to those observed pre-partum. There was no effect of severity of ruminal pH drop on blood NEFA concentration (*P* = 0.91, Figure 2D). Likewise, blood BHB increased within week 1 post-partum (*P* < 0.01); then, started to decline to reach values similar to those found before calving; this variable was unaffected by severity of ruminal pH drop (*P* = 0.57, Figure 2E).

**Figure 3.**
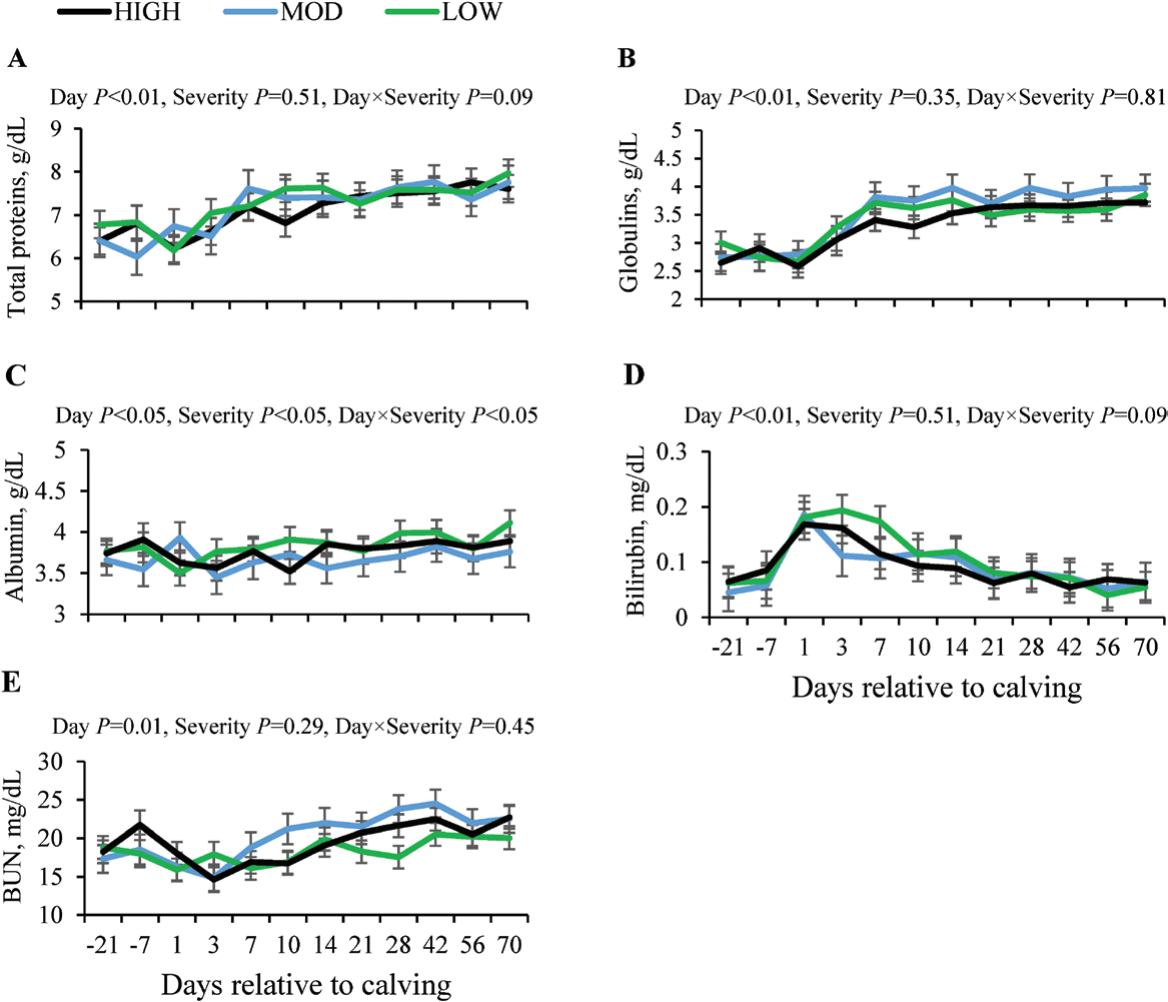
Changes in blood concentrations of total proteins (A), globulin (B), albumin (C), bilirubin (D), and urea nitrogen (BUN, E) relative to calving in primiparous Holstein cows with different severity of ruminal pH drop. Abbreviations: HIGH, high severity; LOW, low severity; MOD, moderate severity.

Blood total protein was lowest from week 3 pre-partum until calving; then, from day 7 post-partum, it increased (*P* < 0.01). However, blood total protein was not affected by ruminal pH drop severity (*P* = 0.51; Figure 3A). Blood content of globulins showed a sharp increase over the first 7 days post-calving (*P* < 0.01), and these increased values were maintained from this time point onwards, with no effect of severity of ruminal pH drop (*P* = 0.35; Figure 3B). Albumin in blood showed a slight increase from week 4 post-calving onwards (*P* < 0.05). In general, LOW cows had greater blood albumin than MOD cows (*P* < 0.05), except at calving (Figure 3C). Bilirubin was highest over the first week post-partum (*P* < 0.01), with no effect of the severity of ruminal pH drop (*P* = 0.51; Figure 3D). The BUN was lowest on days 1 and 3 post-partum, and it increased from day 14 post-partum onwards (*P* < 0.05), but without the effect of severity of ruminal pH drop (*P* = 0.29; Figure 3E).

**Figure 4.**
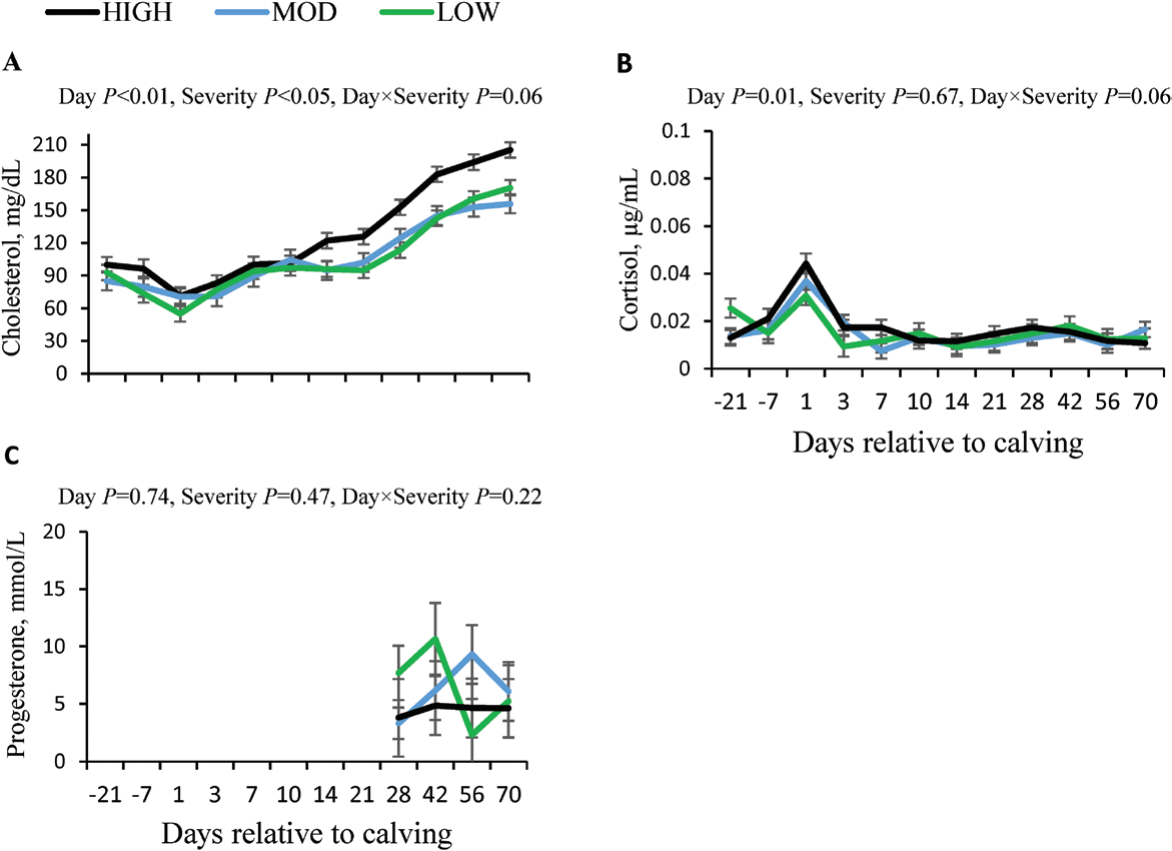
Changes in blood concentrations of cholesterol (A), cortisol (B), and progesterone (C) relative to calving in primiparous Holstein cows with different severity of ruminal pH drop. Abbreviations: HIGH, high severity; LOW, low severity; MOD, moderate severity.

Blood cholesterol reached its lowest concentration at calving, but from day 10 post-partum onwards, a consistent increase was observed (*P* < 0.05). Interestingly, HIGH cows had greater blood cholesterol compared to either LOW or both MOD and LOW (*P* ≤ 0.01; Figure 4A). The higher cholesterol was especially clear from day 14 of lactation onwards; during this time, cholesterol was on average 34 mg/dL greater in HIGH cows than in LOW and MOD. Blood cortisol increased at calving (*P* < 0.01), but it decreased on day 3 of lactation to values similar to those found before calving, and these levels were maintained until day 70 of lactation, with no effect of severity of ruminal pH drop (*P* = 0.67; Figure 4B). Progesterone in blood, measured on days 28, 42, 56, and 70 of lactation, was not affected by day relative to calving (*P* = 0.74) or severity of ruminal pH drop (*P* = 0.47; Figure 4C).

**Figure 5.**
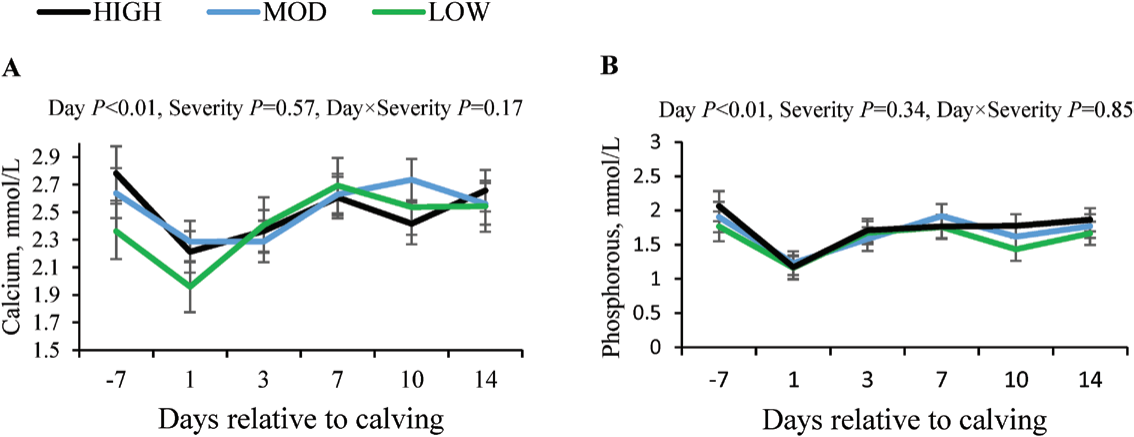
Changes in concentrations of blood calcium (A) and phosphorous (B) relative to calving in primiparous Holstein cows with different severity of ruminal pH drop. Abbreviations: HIGH, high severity; LOW, low severity; MOD, moderate severity.

Blood calcium and phosphorous (Figure 5A,B) reached the lowest levels at calving, but from day 3 post-calving, these variables increased, and levels were maintained from this day onwards (*P* < 0.01). There was no effect of severity of ruminal pH drop on both blood calcium and phosphorous levels (*P* ≥ 0.34).

### Reproductive parameters

The intervals of calving to first service (on average 78 days), calving to conception (on average 105 days), calving interval (on average 382 days), and the number of services to successful conception (on average 1.9) were not affected (*P* ≥ 0.24) by the severity of ruminal pH drop (Table 4).

**Table 4.** Reproductive parameters regarding the second conception in Holstein cows with difference severity of ruminal pH drop

	Ruminal pH drop severity^1^			SEM	*P*-value
	HIGH	MOD	LOW		
Calving to first service, days	77	83	74	6.82	0.60
Calving to conception, days	114	118	82	17.1	0.24
Services to conception, no.	2.11	2.33	1.33	0.59	0.36
Calving interval, days	396	381	370	21.1	0.58

^1^HIGH: high severity of ruminal pH drop; MOD: moderate severity of ruminal pH drop; LOW: low severity of ruminal pH drop.

## Discussion

Previous findings from this project had shown that cows with greater severity of ruminal pH drop had increased DMI and ruminal glucogenic precursors (Hartinger et al., 2024). Thus, we evaluated whether the greater severity of ruminal pH drop would offset the benefits of increased DMI and rumen glucogenic precursors in those cows, which would result in no increase in milk yield and no change in blood glucose, though with lower lipogenic precursors (less ruminal acetate) supply for de novo lipogenesis of body fat and in the udder. All cows were fed the same close-up diet and then gradually transitioned to the same high-concentrate diet and were evaluated up to week 10 in milk. The close-up diet contained 32% concentrate (35.7% NFC and 25.4% peNDF_>8_). Then, the ration changed within the first week post-partum to a lactation diet, which contained 60% concentrate, 40.2% NFC, and 14.4% peNDF_>8_. Despite this change, the LOW cows experienced little shift of ruminal pH, as opposed to the HIGH group where cows experienced the greatest ruminal pH change (Hartinger et al., 2024). For example, in HIGH cows, the time below pH 5.8 increased (*P* < 0.01) from 350 to 446 ± 43.4 min/d from the pre-partum diet to the lactation diet. In addition, the area below pH 5.8 increased (*P* < 0.01) from 56.9 to 81.6 ± 10.8 pH × min/d from the pre-partum diet to the lactation diet. The differences and variability of pH among cows may be due to differences in physiological characteristics, microbial activity, or DMI intake, rather than different positions of the pH boli within the reticulo-rumen.

Results from this study showed that HIGH cows had greater milk production, which can be explained by the greater intake of NE_L_ than the cows in the other ruminal pH categories. Thus, the increased supply of energy improved milk production, with an increase of 5.6 kg ECM/d or 17.5 MJ NE_L_/d (considering 3.14 MJ NE_L_/kg ECM; Erdmann et al., 2019), suggesting that more severe drop of the ruminal pH in HIGH group did not offset the supply of glucogenic energy sources, and lactation performance was enhanced. We initially hypothesized that the higher severity of ruminal pH drop would imply an increased passage rate and decline of nutrient digestibility, impairing the nutrient supply of cows, and so offsetting the benefits of increased DMI and rumen glucogenic precursors. We did not measure the ruminal nutrient digestibility or ruminal passage rate in this trial because cows were not cannulated, but the results indicate an improved energy supply as most of the energy to support the greater milk yield may have originated from the greater DMI as well as from increased glucogenic precursors, such as propionate and valerate in HIGH cows (Hartinger et al., 2024), which are two key glucogenic precursors in cattle (Young, 1977). Thus, our data suggest that individual depression of pH, as observed in the HIGH cows, did not negatively affect milk production when cows were fed the same diet. Other authors have previously reported that various severity of ruminal pH drop in cows fed the same diet did not affect milk yield (Mohammed et al., 2012; Gao and Oba, 2014; Humer et al., 2015, 2018; Khiaosa-ard et al., 2018; Nasrollahi et al., 2017). Therefore, it seems that the ruminal pH drop when the cows are fed the same diet did not impair milk performance, which also supports previous reports showing that low ruminal pH does not necessarily translate to decreased milk yield (Kolver and Veth, 2002); except when the ruminal pH drop has been intentionally induced through abrupt dietary change (Khafipour et al., 2009; Chiquette et al., 2015).

The increased energy intake of HIGH cows was also reflected in improved energy balance during the periparturient period compared to both MOD and LOW cows. In fact, cows with a HIGH severity of ruminal pH drop coped better with the change in energy balance, again suggesting that they were in a better energetic status early post-partum than other cows.

The higher feed intake of HIGH cows seems to have increased ruminal fermentation. As shown in the companion paper, the HIGH cows also showed a tendency for increased ruminal total VFA concentration, lowering the pH and increasing the severity of pH drop (Hartinger et al., 2024). In this context, greater ruminal VFA concentration has been reported to favor their absorption (Dijkstra et al., 1993), which may increase the thickness of ruminal mucosa (Steele et al., 2011). Accordingly, in this study, we found greater RMT when feeding the lactation high-grain diet compared to the close-up diet, but with no effect on individual severity of ruminal pH drop.

In contrast to our hypothesis, the changes observed in the ruminal VFA profile, mainly the decrease of acetate in HIGH cows reported previously (Hartinger et al., 2024), did not have adverse effects on milk composition. This seems to be contradictory because acetate is known to be a key precursor of mammary de novo fat synthesis. Thus, one could expect a decreased milk fat content in HIGH cows, which was not observed. However, this may be explained by the fact that a decreased mammary de novo fat synthesis may have been compensated by body lipid mobilization (Rico and Razzaghi, 2023), which was generally high during early lactation and may also have masked the effects on milk fat content. On the other hand, our results agree with previous reports showing no negative impacts of individual severity of ruminal pH drop on milk fat percent or fat yield in cows consuming the same diet, although with different severity of pH drop, with ruminal pH < 5.8 duration ranging from 244 to 654 min/d during early lactation (Mohammed et al., 2012) or with ruminal pH < 5.8 ranging from 10 to 556 min/d during late lactation (Gao and Oba, 2014). In the literature, there are other reports showing a reduction (Nasrollahi et al., 2017) or tendency for reduced (Khiaosa-ard et al., 2018) milk fat content in mid-lactation cows having greater individual ruminal pH drop compared to cows with less severe pH drop while consuming the same ration. It is important to note, however, that compared to the present study, (Nasrollahi et al., 2017) reported much more severe pH drop conditions for the group with lowest ruminal pH (920 min/d of ruminal pH < 5.8, and very low milk fat in general, <3.0%) than our observations in the present study, which may explain the marked negative impact on milk fat content in their study.

Independent of the severity of the ruminal pH drop, there was an increase in blood glucose at calving. Yet, given the extremely high blood glucose, this could also reflect the dextrose supplemented immediately after calving. Under normal conditions, glucose is taken up by the cells for energy production, especially in a state of negative energy balance. However, cows typically develop insulin resistance at calving to spare glucose for milk synthesis. Thus, the similar blood glucose, albeit greater milk lactose yield in HIGH cows, suggests that part of the glucose generated in HIGH cows may have been channeled into milk lactose. As a result, blood glucose did not differ among ruminal pH categories. In this regard, the low levels of insulin post-partum may have also contributed to stimulate lipolysis; otherwise, high blood insulin can inhibit the hormone-sensitive lipase, a key hormone in lipolysis (Koster et al., 2018).

As expected, we observed that blood triglycerides decreased at the start of lactation and blood NEFA increased, reflecting decreased lipogenesis and increased lipolysis (Larsen and Kristensen, 2012), but with no effect of the severity of ruminal pH drop. The blood NEFA may be oxidized and generate ketone bodies. Thus, our results suggest efficient metabolism of ketone bodies because levels of blood BHB did not indicate accumulation at a level that would indicate subclinical ketosis.

Our findings further show that regardless of the severity of the ruminal pH drop, the increase in blood total protein post-partum was largely influenced by globulins. The low blood total proteins at calving may be due to impaired synthesis of proteins or due to translocation of immunoglobulins and other proteins to the mammary gland for colostrogenesis (Grünberg et al., 2011). Blood albumin was not affected, which is beneficial because of its role in the regulation of blood oncotic pressure and because of its role in NEFA transport (Contreras et al., 2010). Other reports have indicated that inflammation around calving may impair the synthesis of proteins involved in the clearance of waste products (Janovick et al., 2023). In this regard, we observed inflammation around calving but independent of the severity of ruminal pH drop (Hartinger et al., 2024). Therefore, our results suggest that the severity of the ruminal pH drop did not worsen the synthesis of proteins involved in the clearance of bilirubin, and was within the normal range (Janovick et al., 2023). On the other hand, the increase in blood proteins and BUN post-partum may be due to the increased protein intake or due to increased amino acid decarboxylation, generating ammonia and conversion to urea (Bobbo et al., 2017).

Interestingly, this study revealed that cows with high severity of ruminal pH drop had greater blood cholesterol, although within the normal range. This difference was especially clear from days 14 to 70 of lactation. Cholesterol is a pivotal molecule that serves as a component of the cell membrane, in lipoproteins (thus the transfer of triglycerides from the liver), or as a precursor of steroid hormones (Hu et al., 2010) and can be synthesized from acetyl CoA (Buhaescu and Izzedine, 2007). Acetyl CoA may be produced from pyruvate generated after glycolysis, from beta-oxidation of fat, or from direct conversion of amino acids to acetyl CoA (Lehninger et al., 2005). Because all cows consumed the same ration at any given time point in the study, the greater levels of cholesterol in HIGH cows may be due to increased feed intake, which increased the supply of glucogenic precursors. Therefore, contrasting our hypothesis, this finding further shows that the greater severity of ruminal pH drop in HIGH cows did not offset the increased DMI and ruminal gluconeogenic precursors in these cows. Rather, the increased supply of glucogenic precursors contributed to glucose formation; then, part of this glucose was degraded generating acetyl CoA, which may have served for cholesterol synthesis.

Cholesterol can be used to synthesize various steroid hormones such as cortisol, a hormone that may impair fertility due to potential negative effects on luteinizing hormone and estradiol (Wise et al., 1988). Cholesterol may also be used to synthesize progesterone, which plays a crucial role in fertility (Häussler et al., 2022). In this study, however, despite the increment of cholesterol in cows with high severity of ruminal pH drop, no changes were found in cortisol or progesterone. This agrees with our findings on reproductive variables for the second gestation, which did not differ among ruminal pH categories. However, it should be noted that the number of cows and the overall sample size of this study were relatively small for the evaluated parameters; future studies including a larger sample size may overcome this limitation.

We found that the severity of ruminal pH drop did not affect the homeostasis of calcium and phosphorous. Other studies have revealed inverse correlations between acute phase response and plasma calcium in primiparous cows experiencing severe ruminal pH drop with diets containing up to 45% NFC (Zebeli et al., 2010). In this study, there is no indication that cows experienced an inflammatory response due to ruminal pH drop severity as indicated by levels of haptoglobin and serum amyloid A reported in the companion manuscript (Hartinger et al., 2024). It is therefore possible that the lack of an effect of severity of ruminal pH drop on plasma calcium can be explained by the fact that the lower pH did not interfere with calcium metabolism or with systemic inflammation. It should also be mentioned that both starch and NFC levels in our study were lower than in previous studies (Zebeli et al., 2010), contributing to the different responses of ruminal pH on plasma calcium. Additionally, cows in this study had a gradual and slow increase of NFC during the first week post-partum. This was reflected in good health status during transition to early lactation, independent of individual ruminal pH.

It is plausible that a longer time with low ruminal pH is needed to observe an impairment in lactation performance and health of cows. For instance, the low ruminal pH was associated with a tendency for lameness after the study had finished (Kofler et al., 2023), indicating that the effects of low ruminal pH on lactation performance and health need to be evaluated during the entire lactation. In addition, results suggest that some of the effects of individual ruminal pH drop are rapid, such as changes in VFA profile (Hartinger et al., 2024), as well as increased performance, but other effects are delayed (i.e., claw health; Kofler et al., 2023).

The currently available wireless indwelling systems represent an acceptable approach to measure ruminal pH. However, one of the challenges when using such systems is the lack of specific calibration protocols for some of the brands. In addition, when using non-cannulated animals, periodic calibrations cannot be performed, which otherwise could correct a potential drift. Furthermore, when the pH systems are orally dosed, the pH boli usually stay in the reticulum, thus, not necessarily representing actual ruminal pH (Dijkstra et al., 1993).

Regarding the classification of animals according to ruminal acidification, it is worth noting that other investigators have reported a clustering approach using not only ruminal pH data but also feed intake, blood metabolites, and lactation data because of the interconnection among those parameters (Villot et al., 2018; Mensching et al., 2021). Additionally, in studying the effects of ruminal acidification in cows, future research should consider evaluating the effects on milk fatty acid profile as well because milk fatty acid synthesis may be associated with ruminal pH level (Fukumori et al., 2021).

## Conclusion

Taking results from this report and those in the companion papers, our study showed that primiparous cows consuming a diet with the same ingredient and chemical composition can develop different severities of ruminal pH depression. This may even occur when consuming a lower-grain close-up diet before parturition and becomes stronger early postpartum. In addition, results showed that the higher individual severity of ruminal pH drop (for the level observed in this trial) was not associated with negative effects on lactation performance during the first 10 wk in milk in primiparous cows fed a diet with 40% NFC and 14.4% peNDF_>8_. It seems that during this period, the cows adjusted well with the changes occurring in ruminal metabolism due to individual ruminal pH drop severity, especially the acetate yield. Cows also seemed to benefit from rumen glucogenic precursors and did not show signs of systemic inflammation or liver damage due to stronger pH depression, but liver enzymes increased in all ruminal pH categories with advanced duration on the lactation diet. Future research should also evaluate the persistence of individual ruminal pH drops of cows in a larger sample size, across lactations and under more extreme dietary challenge conditions.

## Supplementary Material

skae390_suppl_Supplementary_Figure_S1

## Abbreviations

ADFom: ash-free acid detergent fiber
aNDFom: amylase-treated, ash-free neutral detergent fiber
BCS: body condition score
BFT: back fat thickness
BHB: beta-hydroxybutyrate
BUN: blood urea nitrogen
BW: body weight
MUN: milk urea nitrogen
NEFA: non-esterified fatty acids
NEL: net energy for lactation
NFC: non-fiber carbohydrates
OM: organic matter
peNDF: physically effective NDF
PSPS: penn state particle separator
RMT: rumen mucosa thickness
SCC: somatic cell count
TMR: total mixed ration; δ, minimal detectable difference
