# Supplementary material for: Differences in severity of reticulo-rumen pH drop in primiparous Holstein cows fed the same diet during transition and early lactation: effects on performance, energy balance, blood metabolites, and reproduction
Source: J Anim Sci. 2024 Dec 25;103:skae390. doi: 10.1093/jas/skae390 (PMC11757701; doi:10.1093/jas/skae390)
Supplement: skae390_suppl_Supplementary_Figure_S1 [file skae390_suppl_supplementary_figure_s1.docx]

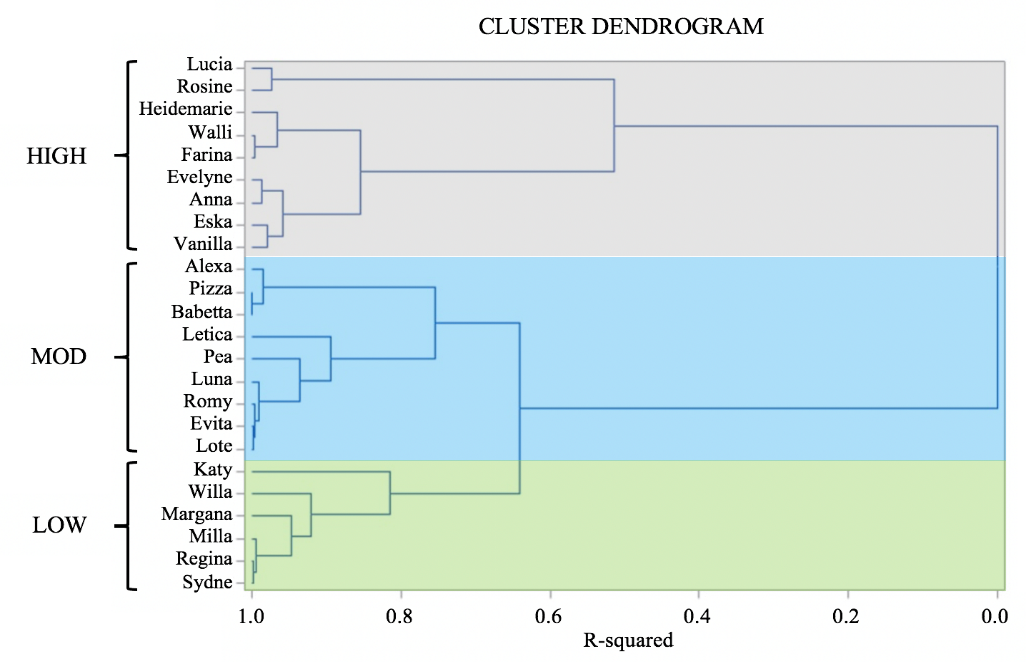


**Supplementary Figure S1**. Hierarchical cluster dendrogram categorizing the cows in high severity of ruminal pH drop (HIGH, n = 9), moderate severity of ruminal pH drop (MOD, n = 9) and low severity of ruminal pH drop (LOW, n = 6).
